# Glucose-6-Phosphate Dehydrogenase Deficiency-Associated Hemolytic Anemia and Methemoglobinemia in a Patient Treated With Hydroxychloroquine in the Era of COVID-19

**DOI:** 10.7759/cureus.15232

**Published:** 2021-05-25

**Authors:** Nicole Laslett, Julianne Hibbs, Max Hallett, Arezoo Ghaneie, Vlasta Zemba-Palko

**Affiliations:** 1 Hematology/Oncology , Lankenau Medical Center, Wynnewood, USA; 2 Hematology and Medical Oncology, Alliance Cancer Specialists, Langhorne, USA; 3 Internal Medicine, Catholic Medical Center, Manchester, USA; 4 Hematology and Medical Oncology, Lankenau Medical Center, Wynnewood, USA; 5 Pathology and Laboratory Medicine, Lankenau Medical Center, Wynnewood, USA

**Keywords:** glucose-6-phosphate dehydrogenase (g6pd), sars-cov-2, covid-19, coronavirus, hydroxychloroquine, methemoglobinemia, hemolytic anemia

## Abstract

Glucose-6-phosphate dehydrogenase (G6PD) deficiency is the most common enzymatic disorder of red blood cells worldwide. The severity of hemolytic anemia varies among individuals with G6PD deficiency, depending on the genetic variant in the G6PD gene; this makes the diagnosis of the condition more challenging in some cases. In this report, we present a case of severe hemolytic anemia and methemoglobinemia in a patient with G6PD deficiency who had been exposed to hydroxychloroquine prescribed for severe acute respiratory syndrome coronavirus 2 (SARS-CoV-2) infection. To the best of our knowledge and based on a literature search, this is one of the first case reports in the literature about hemolytic crisis and methemoglobinemia in a patient with critical illness due to severe coronavirus disease 2019 (COVID-19) who was exposed to hydroxychloroquine. It is critical for physicians and caregivers to recognize the effects of oxidative stressors such as hydroxychloroquine, particularly in this era of the COVID-19 pandemic and in regions with a high prevalence of G6PD deficiency, for the appropriate management of this unique subset of patients.

## Introduction

Glucose-6-phosphate dehydrogenase (G6PD) deficiency is an inherited disorder caused by a genetic defect in the red blood cell enzyme G6PD, affecting around 400 million people worldwide [1]. This enzyme generates nicotinamide adenine dinucleotide phosphate (NADP+) in the reduced form (NADPH) and protects red blood cells from oxidative injury. G6PD deficiency is most commonly reported in the tropical and subtropical zones of the Eastern Hemisphere, correlating with regions where malaria was once endemic [2]. In the steady-state, individuals remain asymptomatic with no hemolysis. However, episodes of hemolysis may be triggered by medications, certain food products, and acute illnesses, especially infections. Hydroxychloroquine, an aminoquinoline, is one medication that is known to cause hemolytic anemia in patients with G6PD deficiency. The drug is widely used in the treatment of malaria and rheumatic disease and has been suggested as an effective treatment for coronavirus disease 2019 (COVID-19) [3]. Patients with G6PD deficiency may be more vulnerable to severe acute respiratory syndrome coronavirus 2 (SARS-CoV-2) infection as G6PD-deficient lung cells infected with human coronavirus 229E result in increased viral production and replication compared with normal cells [4]. The increased susceptibility to infection and hemolysis with secondary tissue hypoxia may result in higher disease severity and even death [5].

## Case presentation

**Figure 1:**
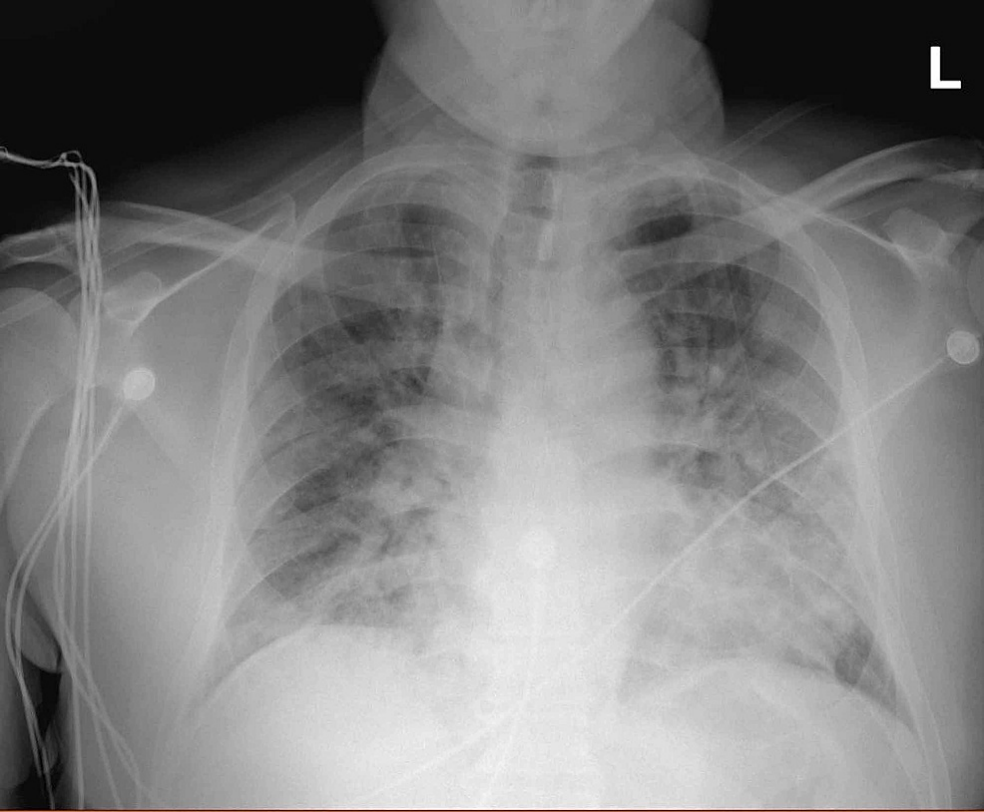
Chest X-ray of the patient The image shows moderate bilateral opacities without effusion

A 60-year-old African American male with G6PD deficiency presented to the emergency department (ED) with two weeks of fever and dizziness. He also reported mild shortness of breath that had started several days prior to the presentation, nausea, an episode of vomiting, and decreased appetite. After the initial evaluation, he appeared to be in moderate respiratory distress and febrile to 101.5 °F, requiring non-invasive bilevel positive airway pressure. Chest X-ray revealed moderate bilateral airspace opacities without pleural effusions (Figure 1). COVID-19 nasopharyngeal polymerase chain reaction (PCR) testing returned positive. The patient required intensive care unit (ICU) care for increasing oxygen requirements. Shortly after his transfer to the ICU, he required intubation for acute hypoxic respiratory failure. Laboratory analysis revealed acute kidney injury, electrolyte abnormalities, high anion gap metabolic acidosis and lactic acidosis, and elevated inflammatory markers with normal blood counts. He was started on antibiotics for presumed community-acquired pneumonia and hydroxychloroquine (800 mg on hospital day one, 400 mg on hospital day two) for severe COVID-19 infection. His hospital course was complicated by pneumothorax and severe hypoxemia requiring prone ventilation and chest tube placement.

**Figure 2:**
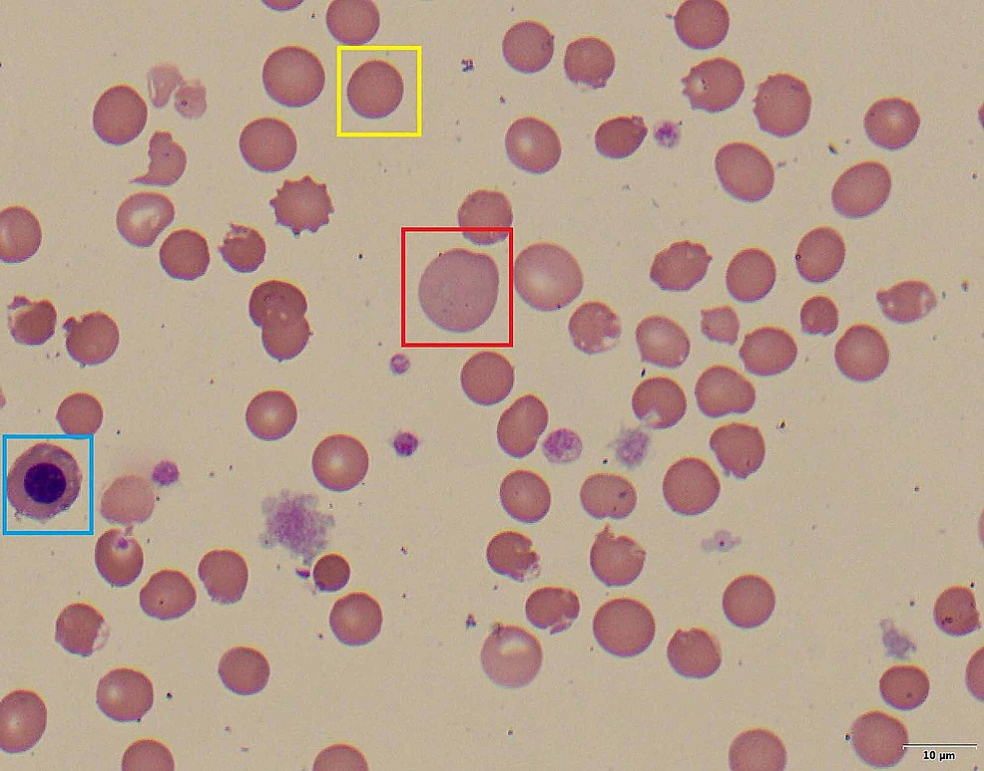
Peripheral blood smear (100x) - image 1 Peripheral blood smear showing a nucleated red blood cell (blue box), spherocyte (yellow box), and reticulocyte (red box)

**Figure 3:**
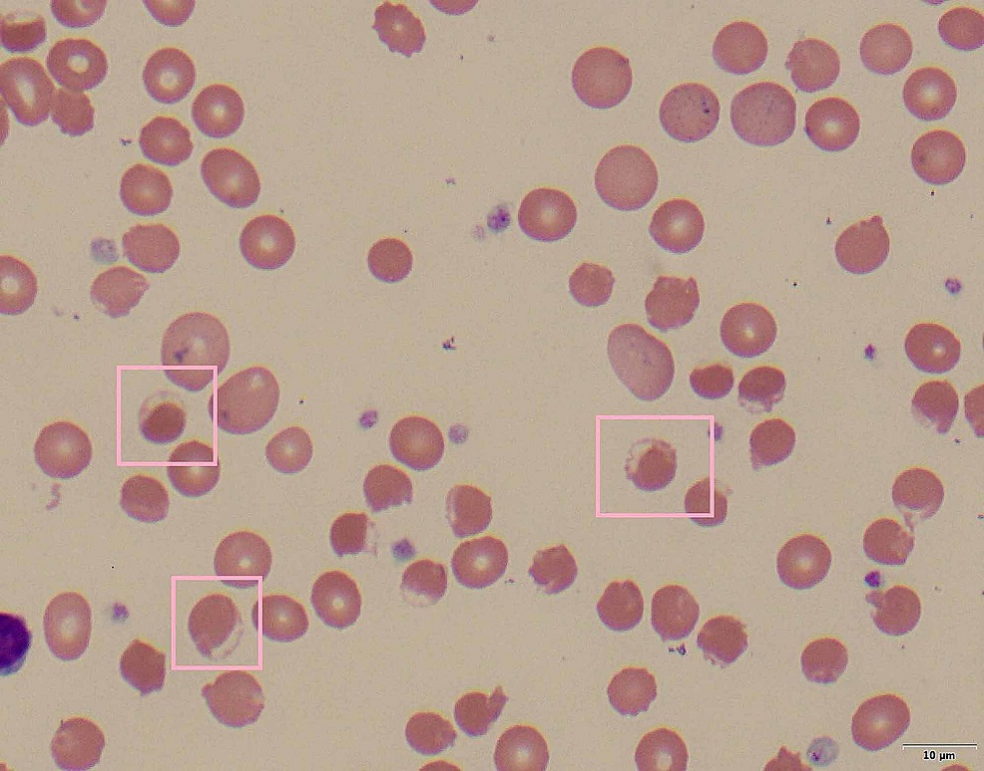
Peripheral blood smear (100x) - image 2 Peripheral blood smear showing hemighost cells (pink boxes)

**Figure 4:**
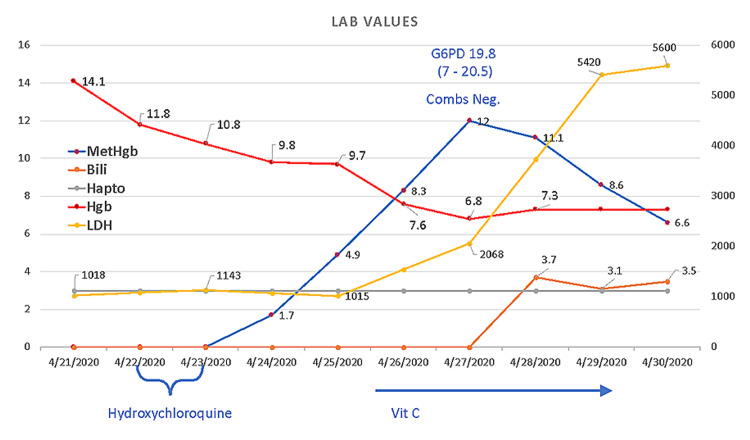
Timeline of blood parameters of hemolysis and methemoglobin levels in our patient G6PD: glucose-6-phosphate dehydrogenase; MetHgb: methemoglobin; Bili: bilirubin; Hapto: haptoglobin; Hgb: hemoglobin; LDH: lactate dehydrogenase; Vit C: vitamin C

On hospital day six, discordance between oxygen saturation (SpO_2_, 88%) and partial pressure of arterial oxygen (PaO_2_, 172 mmHg) was noted. Labs were significant for progressive anemia with hemoglobin of 14.1 g/dL on day one to 6.8 g/dL on day seven, methemoglobin of 1.1% on day four to 12.0% on day seven, lactate dehydrogenase (LDH) of 5,420 on day nine, haptoglobin of <3 mg/dL on day seven, a corresponding rise in bilirubin from 1.8 mg/dL on day one to 5.8 mg/dL on day seven, and a negative direct antiglobulin test (DAT). Peripheral blood smear showed early red cell precursors and hemighost cells (Figures 2, 3). G6PD level was found to be 19.8 U/g Hgb (7.0-20.5 U/g Hgb) on hospital day seven. This was suggestive of methemoglobinemia and severe hemolytic crisis due to underlying G6PD deficiency in the setting of hydroxychloroquine administration and severe COVID-19 illness. The patient was started on vitamin C, folic acid, and supportively transfused with blood products (Figure 4). Despite improved methemoglobin levels with vitamin C therapy, the patient had hemodynamic compromise from a suspected intra-abdominal process, and he subsequently expired.

## Discussion

G6PD deficiency is the most frequently encountered abnormality of red blood cell (RBC) metabolism. It is an X-linked disorder with a varying clinical presentation depending on the extent of the inactivation of the affected X chromosome bearing the abnormal gene. Worldwide, more than 300 variants of G6PD deficiency have been described and are categorized by the WHO according to the extent of enzyme deficiency and severity of hemolysis [6]. For example, the G6PD A(-) variant is present in 10-15% of African American males and represents an unstable variant, resulting in a decrease in enzyme activity in aged RBCs. This is in contrast to other G6PD variants, such as G6PD-Mediterranean, which have a reduced catalytic activity and marked instability rendering both reticulocytes and older cells susceptible to hemolysis. Typically, the G6PD A(-) variant does not manifest anemia until it is exposed to an oxidant drug or challenge such as SARS-CoV-2-infection, leading to an episode of acute intravascular hemolysis. This is due to the failure of the RBCs to generate adequate amounts of NADPH, leading to decreased amounts of oxidized glutathione and thereby facilitating susceptibility of erythrocytes to oxidation of hemoglobin by oxidant radicals.

In our case, the patient likely had the G6PD A(-) variant or one similar and did not show signs of hemolytic anemia in the absence of known oxidative stress. In this variant, or the one that is similar, a moderate amount of young RBCs contain G6PD enzymatic activity, and hence elevated reticulocyte counts in the face of an acute, hemolytic episode raises the mean level of erythrocyte G6PD and can render a false-negative test, as seen in this patient. It is hard to determine if the severity of our patient’s illness was due to underlying G6PD deficiency in the setting of COVID-19 infection, or if the administration of hydroxychloroquine was likely the precipitating cause of the DAT-negative, acute, severe hemolytic crisis and the resultant methemoglobinemia with subsequent, refractory hypoxemia in a G6PD-deficient patient. It is important for physicians to be aware of this phenomenon in critically ill patients as well as the potential for false-negative G6PD results.

**Figure 5:**
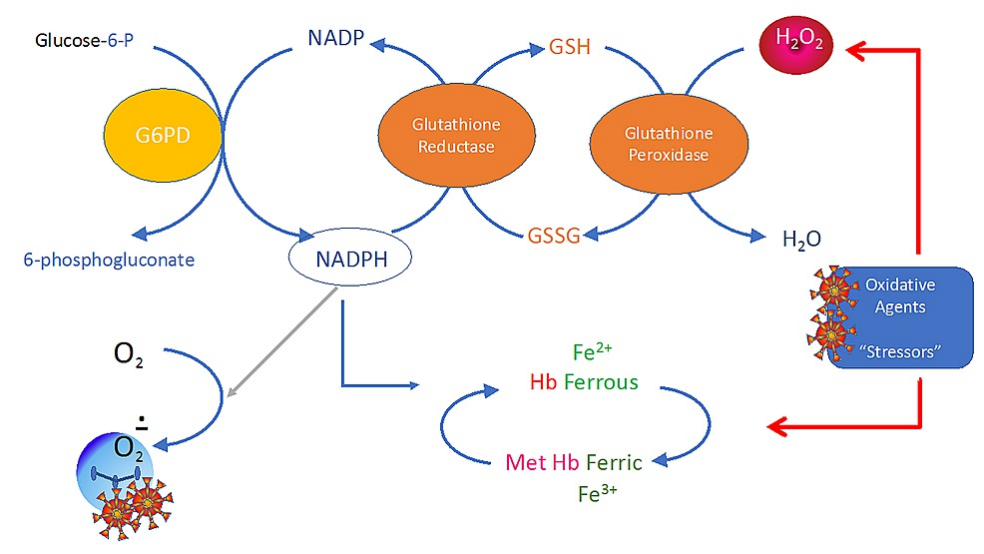
Diagram of oxidative stress on G6PD pathway and conversion to methemoglobinemia G6PD: glucose-6-phosphate dehydrogenase; NADP: nicotinamide adenine dinucleotide phosphate; NADPH: nicotinamide adenine dinucleotide phosphate in the reduced form; GSH: reduced glutathione; GSSG: oxidized glutathione

Severe hemolysis due to G6PD deficiency may manifest as methemoglobinemia. Failure to generate adequate NADPH in G6PD-deficient RBCs leads to heme iron in the oxidized ferric state rather than the ferrous state [7]. The resulting hemoglobin, methemoglobin (metHb), cannot carry oxygen and develops increased oxygen affinity leading to impaired oxygen delivery (Figure 5), a left shift in the oxygen-hemoglobin dissociation curve, and secondary tissue hypoxia [8]. Normal people do generate metHb, but in very low levels (range: 0.5-3.0%) [9]. Our patient’s discrepancy between SpO_2_ and PaO_2_ with elevated levels of methemoglobin was indicative of this process. Typically, levels above 30% can pose life-threatening complications, but levels less than 30% can be equally consequential with underlying hypoxia, such as in COVID-19 infection, as seen in our patient.

Treatment approaches generally involve the cessation of the offending agent and supportive care for G6PD deficiency. In methemoglobinemia, methylene blue and ascorbic acid are accepted as standard forms of therapy. However, methylene blue is contraindicated in G6PD deficiency. The reduction of metHb by methylene blue is dependent upon the NADPH pathway generated by G6PD, and its use is not only ineffective in these patients with G6PD deficiency but may worsen hemolysis due to its oxidant potential [10]. In these specific cases, moderate doses of ascorbic acid (300 to 1,000 mg/day orally in divided doses) should be given [1].

## Conclusions

The use of hydroxychloroquine may trigger severe acute hemolytic crisis in G6PD-deficient patients, and there is scarce data on this disease in the literature, and it may pose serious challenges especially during the ongoing COVID-19 pandemic, when hydroxychloroquine is routinely prescribed for affected patients. There are currently no guidelines that mandate G6PD deficiency screening prior to hydroxychloroquine use, and G6PD deficiency may present late in life, especially in areas with mild variants. Due to the worldwide spread of COVID-19, the use of hydroxychloroquine can expose numerous patients with unknown G6PD deficiency to severe hemolytic crisis, particularly in areas where it is most prevalent, such as tropical and subtropical zones of the Eastern hemisphere. Severe hemolysis as a consequence of critical illness in patients with concomitant G6PD deficiency and COVID-19 may lead to life-threatening methemoglobinemia. Traditional treatment with methylene blue can be even more detrimental in this particular patient population. Physicians should be aware of these potential risks and consequences, especially given the current worldwide transmission of SARS-CoV-2 and the devastating consequences of severe illness in patients with G6PD deficiency and severe hemolytic anemia.
